# Intermetallic Growth and Interfacial Properties of the Grain Refiners in Al Alloys

**DOI:** 10.3390/ma11040636

**Published:** 2018-04-20

**Authors:** Chunmei Li, Nanpu Cheng, Zhiqian Chen, Zhongjing Xie, Liangliang Hui

**Affiliations:** Faculty of Materials and Energy, Southwest University, Chongqing 400715, China; cheng_np@swu.edu.cn (N.C.); chen_zq@swu.edu.cn (Z.C); xzjlms13@email.swu.edu.cn (Z.X); hll0237@email.swu.edu.cn (L.H.)

**Keywords:** intermetallics, interface, first-principles, elastic properties, Griffith rupture work

## Abstract

Al_3_TM(TM = Ti, Zr, Hf, Sc) particles acting as effective grain refiners for Al alloys have been receiving extensive attention these days. In order to judge their nucleation behaviors, first-principles calculations are used to investigate their intermetallic and interfacial properties. Based on energy analysis, Al_3_Zr and Al_3_Sc are more suitable for use as grain refiners than the other two intermetallic compounds. Interfacial properties show that Al/Al_3_TM(TM = Ti, Zr, Hf, Sc) interfaces in I-ter interfacial mode exhibit better interface wetting effects due to larger Griffith rupture work and a smaller interface energy. Among these, Al/Al_3_Sc achieves the lowest interfacial energy, which shows that Sc atoms should get priority for occupying interfacial sites. Additionally, Sc-doped Al/Al_3_(Zr, Sc) interfacial properties show that Sc can effectively improve the Al/Al_3_(Zr, Sc) binding strength with the Al matrix. By combining the characteristics of interfaces with the properties of intermetallics, the core-shell structure with Al_3_Zr-core or Al_3_Zr(Sc1-1)-core encircled with an Sc-rich shell forms.

## 1. Introduction

Experimental studies [1,2] have repeatedly shown that Al_3_Ti and Al_3_Zr particles act as the heterogeneous nuclei and dramatically refine the grains of the α-Al matrix. Recent investigations indicate that Al_3_Sc and Al_3_(Zr, Sc) are also perfect substitutions as grain refiners for Al-Zn-Mg alloys [3,4,5]. A fine-grained Al-6.10%Mg-0.30%Mn 610.25%Sc-0.1%Zr (wt %) alloy [3] shows superior superplastic behavior while subjecting the asymmetrical rolling, which can be ascribed to the fine (sub)grains which may slow down the rate of cavity growth and the stable coherent Al_3_(Sc_1-x_Zr_x_) nano-particles that can ensure a good stability of the fine-grained structure during superplastic deformation. Additionally, in the Al-5.70Zn-1.98Mg-0.35Cu-0.25Sc-0.10Zr alloy [4], Al_3_(Sc_1-x_Zr_x_) nano-particles play an important role in accelerating the cooperative grain boundary deformation and affect the dynamic softening deformation mechanism of Al-Zn-Mg alloys. Besides, the Sc and Zr additions to the Al-Cu-Mg alloy [5] could strongly inhibit recrystallization, refine grain size, impede the segregation of the Cu element along the grain boundary, and increase the spacing of grain boundary precipitates. All these experimental results confirm the importance of refiners. However, choosing the optimized element for micro-alloying aimed at Al alloys’ grain refinement and judging the nucleation behavior of Al_3_TM(TM = Ti, Zr, Hf, Sc) and Al_3_(Zr, Sc) in the Al matrix is clearly based on the intrinsic elastic and thermodynamic properties of the intermetallics of Al_3_TM(TM = Ti, Zr, Hf, Sc) and the interfacial properties of them joining with the Al matrix.

In view of the limitation of experimental techniques, the first-principles approach has been extensively adopted to investigate the properties of the strengthening intermetallics [6], such as Al_3_(Ti_x_V_1-x_) [7], Al_3_Zr [8], and Al-TM (TM = Ti, Zr, and Hf) systems [9]. Besides the properties of the interfaces, precipitates with the Al matrix have also been studied to dissect the strengthening and toughening mechanisms of the precipitates in the matrix, such as the Al/Al_3_Ti interface [10] and the Al/Al_3_Sc [11] interface. Nevertheless, the energy and elastic properties of Al_3_TM(TM = Ti, Zr, Sc) and Al_3_(Zr, Sc) phases, as well as the interfacial modes of them with the Al matrix, have not been systematically studied. Because of the pivotal significance of the grain refiners’ properties in practical applications, we focus on the theoretical investigations of the intermetallics’ properties. We target the interfacial properties between them and the Al matrix. These are done to determine the desirable component of the grain refiner and judge the preferable doping ratio of Sc atoms.

This paper consists of four parts. Section 2 describes the computational methods. Section 3 presents the formation enthalpies, elastic anisotropies, and electronic structures of Al_3_TM(TM = Ti, Zr, Sc) and Al_3_(Zr, Sc) intermetallics, with the interfacial properties of Al/Al_3_(Zr, Sc). Finally, Section 4 provides a brief summary and conclusion of this paper. The accuracy of our first-principles calculations is assessed by comparing the theoretical calculation results with the experimental values for lattice parameters, elastic properties, and formation energies.

## 2. Method and Models of Calculation

### 2.1. Models Construction

**Figure 1:**
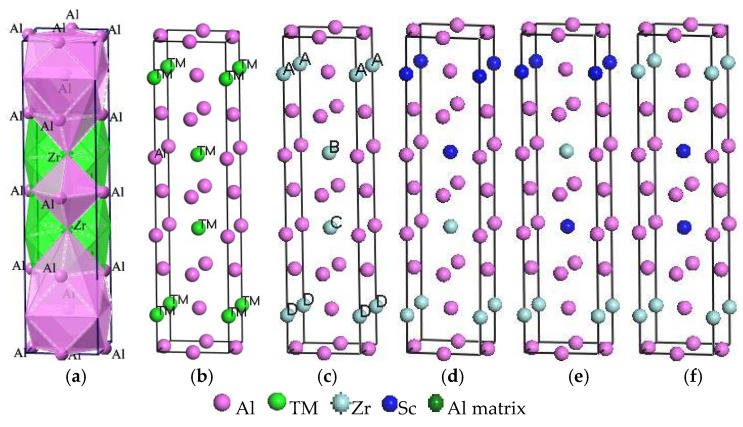
Crystal models. (**a**) Polyhedral model of Al_3_Zr; (**b**) Al_3_TM (TM = Ti, Zr, Hf, Sc); (**c**) Al_3_Zr(Al:Zr = 12:4); (**d**) Al_3_Zr(Sc2-1) (Al:Zr:Sc = 12:2:2); (**e**) Al_3_Zr(Sc2-2) (Al:Zr:Sc = 12:2:2); (**f**) Al_3_Zr(Sc2-3) (Al:Zr:Sc = 12:2:2).

In this study, Al_3_TM(TM = Ti, Zr, Hf, Sc) and Al_3_(Zr, Sc) phases combined with the interfaces of the Al matrix have been investigated by using the first-principles method on the basis of the DFT plane-wave method [6]. Figure 1a,b shows the unit cell of Al_3_TM(TM = Ti, Zr, Hf, Sc) phases, where Figure 1a is a polyherdral model for Al_3_Zr, while Figure 1b represents only the atomic site occupations. Al_3_TM(TM = Ti, Zr, Hf, Sc) phases all crystallize in the I4/MMM space group (No. 139) with a tetragonal crystal system and Al crystallizes in the FM-3M space group (No. 225) with a cubic crystal system. The corresponding lattice parameters, volumes of unit cells, crystal structures, and space groups are shown in Table 1. The substitutional unit is modeled in a 2 × 2 × 1 supercell with periodic boundary conditions, which is shown in Figure 1. In order to gain the efficient doping ratio of Sc in Al_3_Zr, the doping ratio increases from 1/16 to 15/16. Series of models are built and calculated combining the doping ratio at the doping site. Based on these former calculations, the influence of the Sc-doping ratio on the crystal formation can be gained. Then, with the purpose of decreasing the quantity of the calculation, the smaller calculation units shown in Figure 1c–f are built. Once again, the Sc-doping ratio and site are taken into account.

**Figure 2:**
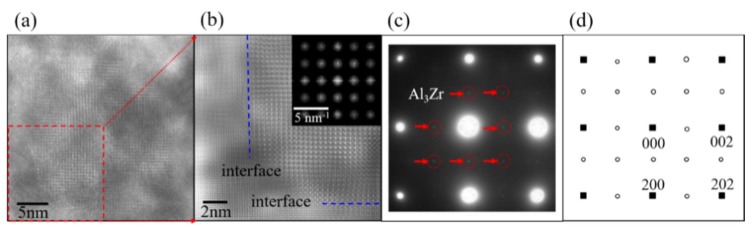
HRTEM and SAED images of Al_3_Zr in Al matrix ([100] zone axis). (**a**) HRTEM image of Al_3_Zr in Al matrix; (**b**) enlarged micrograph of Al_3_Zr in red frame of (**a**), with its fast Fourier transformation (FFT) as inset, where blue dashes present the major interfaces between particle and matrix; (**c**) SAED pattern of (**a**); (**d**) schematic representation of SAED pattern with projection along [100] zone axis, where small solid squares stand for Al matrix, and small open circles represent Al_3_Zr.

Figure 2 shows the high-resolution transmission electron microscopy (HRTEM), together with the selected area electron diffraction (SAED) image of Al_3_Zr in the Al alloy at a solid-solution state. This experimental Al alloy (Al-Zn0.078-Mg0.018-Cu0.015-Zr0.001) (wt %) was prepared by semi-continuous casting, and step-homogenized at 430 °C for 18 h and then at 467 °C for 4 h. After that, the homogenized sample was subjected to solution and quenching treatments. Based on the SAED image shown in Figure 2c, it can be deduced that phase Al_3_Zr is coherent with the Al matrix, with its [1] and [010] crystallographic orientations parallel to [001] and [010] crystallographic orientations of the Al matrix. Combining the HRTEM image of Al_3_Zr precipitated in the Al matrix with other research results [6,7,8,9], it can be judged that the Al_3_Zr{001}/Al{001} interface mode is one of the dominating interface modes of Al_3_Zr phase in the Al matrix. In addition, the diameter of the Al_3_Zr particle in the Al matrix is always around or larger than 20 nm, as shown in Figure 2a, just as the other work reported in References [1,2,3,4,5]. Therefore, to focus on the property of the interface and simplify the calculated unit cell, the interfaces between Al_3_Zr and the Al matrix are treated as flat and periodic during first-principle calculations. What is more, because of the best match of the Al_3_Zr (001) crystallographic plane with the Al (001) crystal face according to the lattice parameters shown in Table 1, the interface models were built as Al_3_Zr(001)/Al(001) interfaces perpendicular to [001] crystal orientation.

**Figure 3:**
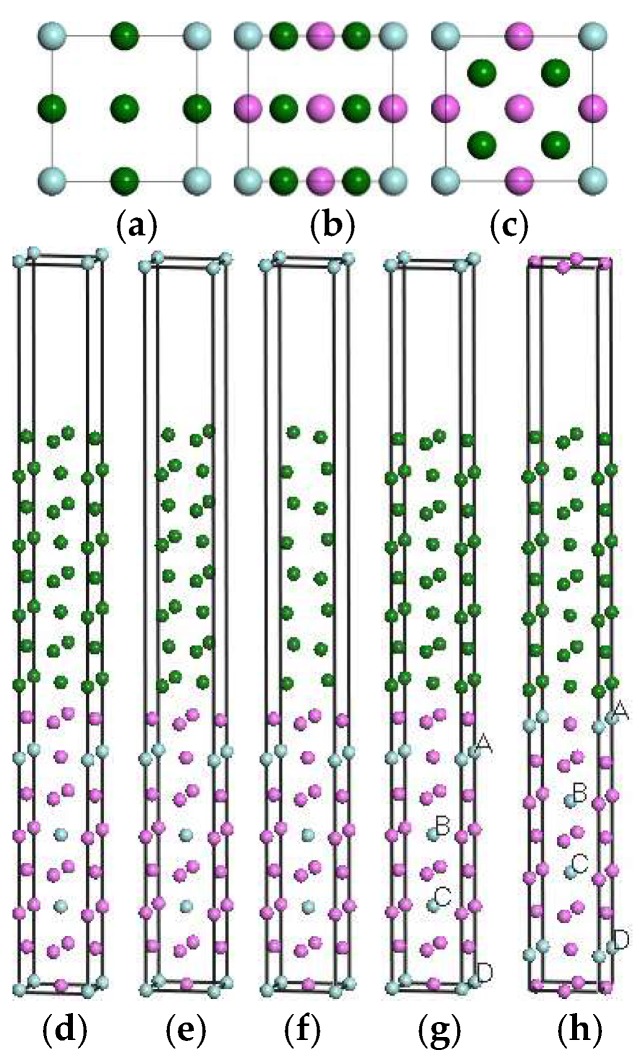
Interfacial stacking models paralleling to (001) plane. (**a**) top view of top-site model; (**b**) top view of bridge-site model; (**c**) top view of central-site model; (**d**) stereogram of top-site model; (**e**) stereogram of bridge-site model; (**f**) stereogram of central-site model; (**g**) Al/Al_3_TM(I-ter); (**h**) Al/Al_3_TM(D-ter).

To determine the most preferable interface mode, the initial calculated models were built with different terminals and different jointing (stacking) modes, namely the interfaces docking with different surface atoms and relative positions. In this work, the interface modes are built in two different forms. One is signed as the I-ter mode with indirect Al/Al_3_TM jointing (Figure 3g), in which mode the Al matrix is jointed with the Al atoms layer in Al_3_Zr but not directly with the layer including the Zr atom. The other is the D-ter mode with direct Al/Al_3_TM binding (Figure 3h), in which mode the Al matrix is combined with the layer including the Zr atom in Al_3_Zr directly. In the I-ter mode, the interfacial properties of the adjacent Al/Al interface are discussed by comparing them with D-ter, which aims to distinguish its internal cohesion between the Al layer and Al_3_TM with the interfacial bonding energy in the D-ter mode. Three stacking modes including top-site stacking, bridge-site stacking, and central-site stacking are considered. The top views and side views of them are shown in Figure 3a–f, respectively. Convergence tests show that the further increase of the layers has no impact on the calculation results of interfacial properties on the condition that the thickness of the two substances in the interface is larger than five layers. Taking into account the different doping sites in Figure 3g,h, the influence of Sc-doping on the interfacial properties is investigated. As shown in Figure 3, the sites of Zr atoms in crystal (Figure 1c) are equivalent, but they are nonequivalent in interfacial models (Figure 3g,h).

Crystal and interface models of Sc-doped Al_3_(Zr, Sc) are separately named Al_3_Zr (Sc n-m) and Al/Al_3_Zr (Sc n-m), respectively. The letter “n” represents the number of doped Sc elements in the calculation unit shown in Figure 1 and Figure 3. The letter “m” is set on behalf of the doping sites of Sc. Models of Al_3_Zr(Sc 2-1), Al_3_Zr(Sc 2-2), and Al_3_Zr(Sc 2-3) are shown in Figure 2c–e, corresponding to an Sc-doping ratio of 1/2 with different Sc-doping sites at A & B, A & C, and B & C, respectively. The structures with other doping ratios in the calculation unit cell all only have one structure each. However, interface models can be distinguished. Al/Al_3_Zr(Sc 1-1), Al/Al_3_Zr(Sc 1-2), and Al/Al_3_Zr(Sc 1-3) are all on behalf of the models with a 1/4 Sc-doping ratio, but correspond to different doped sites at A, B, and C, respectively. Al/Al_3_Zr(Sc 2-1), Al/Al_3_Zr(Sc 2-2), and Al/Al_3_Zr(Sc 2-3) are interface models with a 1/2 Sc-doping ratio that correspond to crystal models shown in Figure 2c–e, respectively. Al/Al_3_Zr(Sc 2-4) model is constructed with Al_3_Zr(Sc 2-1) (shown in Figure 2c) cut from the middle section of planes containing A and B. Al/Al_3_Zr(Sc 3-1), Al/Al_3_Zr(Sc 3-2), Al/Al_3_Zr(Sc 3-3), and Al/Al_3_Zr(Sc 3-4) are models with an Sc-doping ratio of 3/4 and correspond to different un-doped sites at D, C, B, and A, respectively.

### 2.2. Energy Calculation Method

All energy calculations are performed by using the pseudo-potential plane-wave method [22] and are implemented through the Cambridge Serial Total Energy Package Program [23]. The electronic exchange-correlation energy is determined by using the generalized gradient approximation of Perdew-Burke-Ernzerh (GGA-PBE) [24]. All crystal structures are fully relaxed with respect to the volume, as well as to all cell-internal atomic coordinates. The convergence of results with respect to energy cutoff and *k*-points [25] is carefully considered. A plane-wave basis set is used with an energy cutoff of 330 eV. The summation over the Brillouin zone for the bulk structures is performed on a Monkhorst-pack *k*-point mesh with spacing of 0.04 nm^−1^ for all calculations. All atomic positions are optimized by using the Broyden-Flecher-Goldfarb-Shanno (BFGS) scheme [26] based on the cell optimization criterion. The convergence is confirmed with the system total energy fluctuation within 5 × 10^−6^ eV, with the force on each atom in the unit cell less than 0.01 eV/Å, with the residual stress of the unit cell lower than 0.02 GPa, and with the tolerance offset lower than 5 × 10^−6^ Å.

A series of first-principles calculations (at 0 K) of the Al_3_TM(TM = Ti, Zr, Hf, Sc) and Al_3_(Zr, Sc) phases, bulk Al, Ti, Zr, Hf, and Sc, and free atom Al, Ti, Zr, Hf, and Sc are calculated to explain the different formations of the intermetallic compounds. The formation enthalpies per atom (*ΔH*) of the Al_3_TM(TM = Ti, Zr, Hf, Sc) and Al_3_(Zr, Sc) phases can be calculated by using the following Equation (1) [27,28]:

(1) $$\Delta H_{A_{n_{1}}B_{n_{2}}}=\frac{1}{n_{1}+n_{2}}[E_{t}^{A_{n_{1}}B_{n_{2}}}-n_{1}E_{s}^{A}-n_{2}E_{s}^{B}]$$

where *E_t_* is the total energy calculated at *T* = 0 K and *E_s_^A^* and *E_s_^B^* are the energies per atom of bulk *A* and *B*, respectively. All atoms are relaxed to their equilibrium geometries. The same potential function has been used in the total energy calculation of the intermetallics and bulk pure metals. The calculated energies per atom of bulk Al, Ti, Zr, Hf, and Sc are −56.42 eV, −1603.07 eV, −1280.95 eV, −408.84 eV, and −1277.17 eV, respectively. Therefore, the formation enthalpies per atom of the intermetallics can be calculated.

The binding energies per atom (*E_b_*) of the Al_3_TM(TM = Ti, Zr, Hf, Sc) and Al_3_(Zr, Sc) phases can be calculated by using Equation (2) [29]:

(2) $$E_{b}^{{}_{A_{n_{1}}B_{n_{2}}}}=-\frac{1}{n_{1}+n_{2}}[E_{t}^{{}_{A_{n_{1}}B_{n_{2}}}}-n_{1}E_{a}^{A}-n_{2}E_{a}^{B}],$$

where *E_a_^A^* and *E_a_^B^* are the energies per atom of free atoms *A* and *B*, respectively. The calculated energies per atom of the free atoms Al, Ti, Zr, Hf, and Sc are −52.66 eV, −1596.38 eV, −1273.70 eV, −401.49 eV, and −1272.62 eV, respectively, and the calculated binding energies are shown in Table 2.

### 2.3. Elastic Properties Calculation Method

By using the Voigt–Reuss–Hill approximation [30], we calculate the polycrystalline elastic properties, bulk modulus, and the shear modulus (shown in Table 3) on the basis of the second-order elastic constants, which are determined by means of linear fitting of the stress-strain curves [30].

(3) $$B=(B_{R}+B_{V})/2,G=(G_{R}+G_{V})/2$$

The tetragonal system has six independent elastic constants: *C_11_*, *C_12_*, *C_13_*, *C_33_*, *C_44_*, and *C_66_*. The Voigt bounds (*B_v_*, *G_v_*) and the Reuss bounds (*B_R_*, *G_R_*) [31] are expressed below.

(4) $$B_{V}=(2C_{11}+2C_{12}+C_{33}+4C_{13})/9$$

(5) $$G_{V}=(2C_{11}-C_{12}+C_{33}-2C_{13}+6C_{44}+3C_{66})/15$$

(6) $$B_{R}=[(C_{11}+C_{12})C_{33}-2C_{13}^{2}]/(C_{11}+C_{12}+2C_{33}-4C_{13})$$

(7) $$G_{R}=\frac{15C^{2}(C_{11}-C_{12})C_{44}C_{66}}{2(C_{11}-C_{12})(2(C_{11}+C_{12})+4C_{13}+C_{33})C_{44}C_{66}+3C^{2}(2C_{44}C_{66}+(C_{11}-C_{12})(C_{44}+2C_{66})}$$

where:

(8) $$C^{2}=C_{33}(C_{11}+C_{12})-2C_{13}^{2}$$

The Young’s modulus and Poisson’s ratio [32] can be calculated by using the equation below.

(9) E=9BG/(3B+G)

(10) ν=(3B−E)/(6B)

### 2.4. Griffith Rupture Work and Interfacial Energy Calculation Method

The Griffith rupture work (*W*) [33] is defined as the energy required per unit area to reversibly separate a bulk material into two semi-infinite bulks with two free surfaces. It is sometimes called the “ideal work of separation.” In the present study, *W* is calculated according to Equation (11).

(11) $$W=-[E_{{\mathrm{Al}}_{3}\mathrm{TM}/\mathrm{Al}}-E_{{\mathrm{Al}}_{3}\mathrm{TM}}-E_{\mathrm{Al}}]/A$$

where *E*_Al3TM_ and *E*_Al_ are the total energies of the Al_3_TM (TM = Ti, Zr, Hf, Sc) and α-Al with free surfaces, respectively. *E*_Al3TM/Al_ is the total energy of the Al_3_TM/Al supercell embedded in Vacuum and *A* is the area of the interface. All systems are calculated under exactly the same conditions (*k* mesh, cutoff energy, etc.). They are all subjected to the same lateral lattice strain set by the underlying lattice. Perpendicularly to the interface, all the atoms are fully relaxed. The Griffith rupture work calculated in this manner gives direct information regarding the strength and bonding of the interface and is taken as a measure for the mechanical stability and chemical bonding strength at the interface [33].

The interfacial energy *γ* is calculated by using the equation below.

(12) $$\gamma =(E_{Al_{3}TM/Al}-\sigma_{Al_{3}TM}A-\sigma_{Al}A-E_{Al_{3}TM}^{Bulk}-E_{Al}^{Bulk})/A,$$

where *E_Al3TM_^Bulk^, E_Al_^Bulk^* corresponds to the total energies of Al_3_TM (TM = Ti, Zr, Hf, Sc) and Al in bulk states and *σ*_Al3TM_ and σ_Al_ are the surface energies of the free surfaces of the Al_3_TM and Al matrix, respectively. All systems are subjected to the same lateral strain imposed by the underlying lattice. The surface energies are calculated relative to the bulk suffering the same strain as the interface.

(13) $$\sigma_{X}=(E_{X}-E_{X}^{Bulk})/2A,$$

where X stands for Al_3_TM or the Al matrix. Combining Equations (2)–(4), the interfacial energy can also be expressed as the following equation:

(14) $$\gamma =\sigma_{\eta^{\prime }}+\sigma_{A^{l}}-W.$$

Equation (5) presents the relationship between *W* and *γ* and shows that the interfacial energy varies in a contradictory way to the work of separation.

## 3. Results and Discussion

### 3.1. Energy

Table 2 shows the calculated formation enthalpies and binding energies per atom of the four intermetallics of Al_3_TM (TM = Ti, Zr, Hf, Sc). The results show that the absolute value of formation enthalpy for the intermetallics decreases in the following order: Al_3_Zr > Al_3_Sc > Al_3_Hf > Al_3_Ti. All the absolute values are higher than the main strengthening phases (MgZn_2_, Al_2_CuMg, Al_2_Cu) in Al-Zn-Mg-Cu alloys [28]. Formation enthalpy is defined as the released or absorbed energy during the reaction, which shows the ease or difficulty for the formation of intermetallics. On the condition that formation enthalpy is negative, a larger absolute value means easier formation of the intermetallic phase. According to this theory, these four intermetallics of Al_3_TM (TM = Ti, Zr, Hf, Sc) can all act as grain refiners for Al alloys. Furthermore, among these four compounds, the formation of Al_3_Zr is easiest during the solidification process under the same circumstance, which is beneficial to its nucleation and contributes to the Al-matrix’s grain refining. Based on the phases’ energy analysis, Al_3_Zr is the most suitable for use as a grain refiner among these four intermetallics.

The binding energy of the intermetallics decreases in the following order: Al_3_Zr > Al_3_Ti > Al_3_Sc > Al_3_Hf. The binding energy represents the strength of atomic bonding and reflects the stability of the phase. Again, Al_3_Zr has the largest binding energy among the four intermetallic phases, which means that the atomic bonding of Al_3_Zr is strongest and most difficult to dissolve. Following heat treatment after being casted, Al_3_Zr is a perfect particle to pin the dislocation and grain boundary that is contributing to hindering grain growth and grain refinement. On the contrary, Al_3_Ti has the lowest absolute value of formation enthalpy and a comparatively higher binding energy. It is the least suitable for acting as a grain refiner among the four intermetallics based on the energy analysis.

### 3.2. Elastic Properties

The calculated elastic results of the four phases are shown in Table 3. The Young’s moduli of these intermetallics decrease in the following order: Al_3_Zr > Al_3_Ti > Al_3_Hf > Al_3_Sc. Al_3_Zr achieves the highest Young’s modulus, which means that this phase can act as a strengthening phase in the Al matrix during deformation. However, according to Pugh’s criterion [44], a material is brittle (ductile) if the *B*/*G* value is less (greater) than 1.75. What is more, a low (high) Possion’s ratio is the representation of brittleness (toughness) of materials [29]. Based on these two criterions, the ductility of the four intermetallics decreases in the following order: Al_3_Hf > Al_3_Sc > Al_3_Ti > Al_3_Zr. As a result, the brittleness of the Al_3_Ti or Al_3_Zr phase implies that their existence may act as a crack initiation point. However, the calculated results show that Sc-doped Al_3_Zr can effectively promote its ductility as Al_3_(Zr, Sc) particles simultaneously with a high Young modulus. Furthermore, Poisson’s ratio *υ* shows the condition of the atomic binding force. When *υ* lies between 0.25 and 0.5, it means that the atomic binding force is a central force. The data in Table 4 show that the atomic binding force of Al_3_TM (TM = Ti, Zr, Hf, Sc) phases is not the central force. Based on the results of elastic properties, Al_3_Hf and Al_3_(Zr, Sc) are more suitable for use as grain refiners than Al_3_Ti.

**Figure 4:**
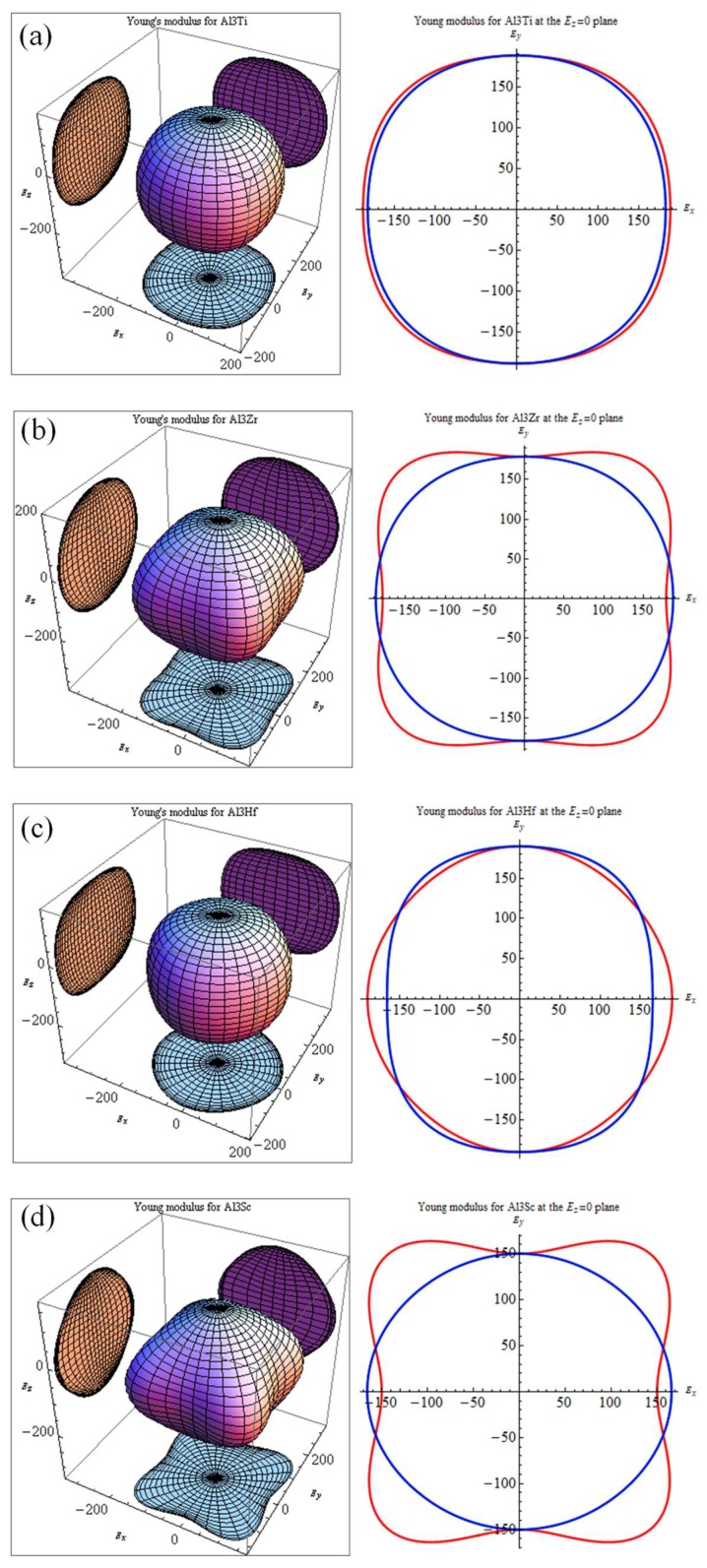

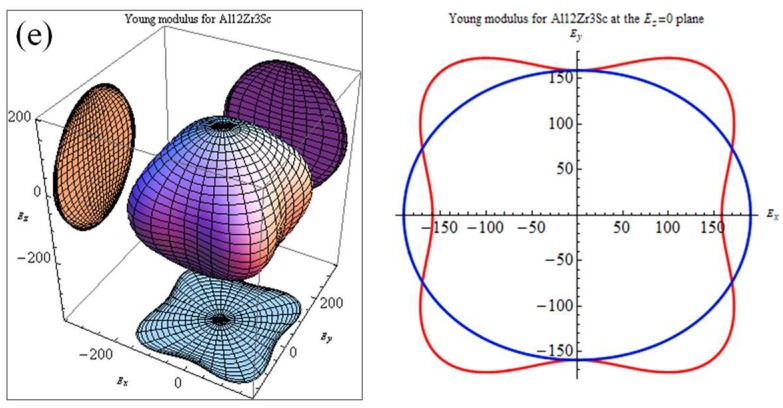
Directional dependences of Young’s moduli for (**a**) Al_3_Ti, (**b**) Al_3_Zr, (**c**) Al_3_Hf, (**d**) Al_3_Sc, and (**e**) Al_12_Zr_3_Sc; projections of the directional dependent Young’s moduli in different planes for Al_3_Ti, Al_3_Zr, Al_3_Hf, Al_3_Sc, and Al_12_Zr_3_Sc. The units are in GPa.

Micro-cracks are easy to induce in materials because of significant elastic anisotropy [49]. Therefore, elastic anisotropy should be calculated to predict the mechanical durability of materials. A 3D curved surface, which represents the dependence of elastic properties on crystallographic directions, can indicate the elastic anisotropy of crystal structures. The Young’s moduli with directional dependence for tetragonal crystal are defined in Equation (15) [50].

(15) $$E_{T}=1/[(l_{1}^{4}+l_{2}^{4})S_{11}+l_{3}^{4}S_{33}+l_{1}^{2}l_{2}^{2}(2S_{12}+S_{66})+l_{3}^{2}(1-l_{3}^{2})(2S_{13}+S_{44})+2l_{1}l_{2}(l_{1}^{2}-l_{2}^{2})S_{16}]$$

where *S_ij_* is the elastic compliance constant and *l*_1_*, l*_2_*,* and *l*_3_ are the directional cosines to the *X*, *Y*, and *Z* axes, respectively. Figure 4 shows the bulk moduli and Young’s moduli with directional dependence of Al_3_TM (TM = Ti, Zr, Hf, Sc) and Al_12_Zr_3_Sc combined with the projection of the calculated elastic moduli on the *XY*, *XZ*, and *YZ* planes.

Theoretically, the curved surface in an isotropic system should be spherical, whereas the deviation from the spherical shape indicates the extent of elastic anisotropy. It is true that the anisotropy of the disoriented precipitation should not be reflected in the macro performance. However, the anisotropy of the intermetallics may determine local strain imposed by external stress and may result in the generation of micro-cracks. Thus, the anisotropy and relative orientation distribution may affect alloy performances. Comparatively, Al_3_Sc shows a more serious anisotropy than Al_3_Zr. As a result, if Sc is added as a grain finer together with the Zr element, the Young’s modulus of Sc-doped Al_12_Zr_3_Sc_1_ with the Sc-doping ratio at 1/4 can show a more favorable isotropy property, which is shown in Figure 4e. This means it may be a better grain refiner than Al_3_Sc [3]. From the elasticity perspective, the simultaneous addition of Zr and Sc is better than adding Sc individually.

### 3.3. Griffith Rupture Work and Interfacial Energy of Al(001)/Al_3_TM(001)

Table 4 tabulates the Griffith rupture work (*W*_ad_), surface energy (σ_suf_), and interfacial energy (γ_int_) of two kinds of interface models with I-ter and D-ter (shown in Figure 3). It was found that only the top-site stacking modes and part of the central-site stacking modes are stable. Additionally, the top-site modes are more stable than the central-site stacking modes. Furthermore, the Griffith rupture work (*W*_ad_) of I-ter models is generally higher than that of D-ter models, which means that the combination mode of Al_3_TM (TM = Ti, Zr, Hf, Sc) with the Al matrix tends to occur more often with I-ter in Al_3_TM intermetallics. Besides, the lower surface energy of the Al_3_TM models with I-ter compared with D-ter (shown in Table 4) also shows that Al_3_TM is more likely to be exposed to an external environment with I-ter based on the lowest energy criterion. The Griffith rupture work of the Al/Al_3_TM interface in I-ter is much larger than that in D-ter modes. In other words, even the bonding strength of the neighboring Al/Al interface is higher than the direct Al/Al_3_TM interface in D-ter modes, which remarkably characterizes the good wetting property or cohesion of the Al(001)/Al_3_TM(001) interface with D-ter. This is consistent with [8]. The Griffith rupture work with I-ter decreases in the following order: Al_3_Sc > Al_3_Zr > Al_3_Hf > Al_3_Ti, which indicates that the binding strength of Al_3_Sc with the Al matrix is higher than the others. Additionally, Al_3_Zr also shows a good performance for combining with the Al matrix. From the interface perspective, it can also be deduced that Al_3_Zr doped with Sc can effectively improve its binding strength with the Al matrix.

Interface energy (γ_int_) is defined as the excess energy per unit area during an interface forming. This is intrinsically caused by the changes of interfacial chemical bonds and structure strain between the two substances constructing the interface. Therefore, the stability of an interface can be assessed by its interface energy. Basically, if both of the materials are more distinct, then the γ_int_ will be larger and the interface will be more unstable [51]. For the two different terminals, the interface energy of the models shows the same increasing sequence as Al_3_Sc < Al_3_Zr < Al_3_Hf < Al_3_Ti. First, this is due to the different lattice deviations of Al_3_TM from Al, which are shown in the same order of Al_3_Sc < Al_3_Zr < Al_3_Hf < Al_3_Ti (as shown in Table 1). Among these interfaces, Al_3_Ti has a positive interface energy, which is mainly caused by the structure mismatch and interfacial strain [51] shown in Table 1. This explains why Al_3_Ti cannot be a favorable grain refiner even though it shows a perfect performance in terms of its elastic properties, which was discussed in Section 3.2. In addition, due to the small absolute value of the interface energy of the Al_3_Hf/Al interface, the driving force to form the Al_3_Hf/Al interface is limited, so the formation of the Al_3_Hf/Al interface is restricted during solidification, which imposes a detrimental effect on its refining ability. In contrast, the negative interface energies of Al_3_Sc and Al_3_Zr with the Al matrix are large enough to affirm their inter-diffusion of the interfacial joint [52]. It is believed that the interface with a negative γ_int_ will provide a driving force to push interfacial atoms diffusing across the interface and bring them into the interfacial alloying, as well as potentially form an interfacial new phase. Therefore, the negative interface energy has significantly more influence on the interfacial structure and morphology. Among the eight interface models, the combination of I-ter Al_3_Sc with the Al matrix has the lowest negative interface energy, which confirms the positive wetting ability of Sc element in the interface. As such, Sc is a favorable additive element for Al_3_Zr to further improve its dispersion precipitation in the Al matrix during solidification, which is beneficial for further refining and strengthening the aluminum alloy.

### 3.4. Sc-Doped Al_3_(Zr,Sc) Phase and Al/Al_3_(Zr,Sc) Interface

#### 3.4.1. Energy of Al_3_(Zr, Sc)

**Figure 5:**
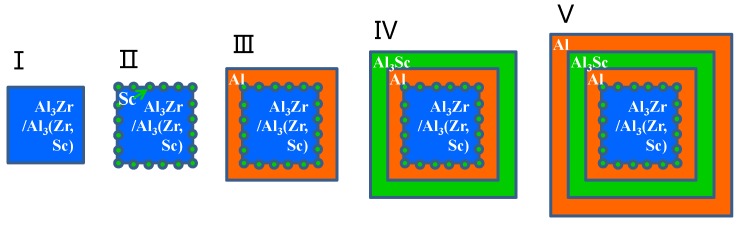
The schematic diagram of core-shell structure forming during solidification process with Al_3_(Zr, Sc) or as nuclei.

The formation enthalpy and binding energy of the Al_3_(Zr, Sc) models defined in Section 2.1 are shown in Table 5. Along with these models, we have calculated the formation energies of Al_3_(Zr, Sc) series with an Sc: Zr ratio from 1:16 to 15:16 by using 2 × 2 × 1 supercell models as stated in Section 2.1 and found that the formation enthalpy of Al_3_(Zr, Sc) was the minimum value with Sc:Zr at 1:3. It is shown that the absolute value of the formation enthalpy of Al_3_Zr(Sc1-1) with the Sc:Zr ratio at 1:3 is higher than that of both Al_3_Zr and Al_3_Sc, which means that Al_3_Zr(Sc1-1) has the highest nucleation driving force. Additionally, Al_3_Zr(Sc1-1) has a higher absolute value of binding energy than other Al_3_(Zr, Sc) models, which leads to a higher melting point and results in a higher precipitation temperature during solidification. This means the Al_3_Zr(Sc1-1) structure is the easiest model to nucleate under the condition of adding Sc and Zr simultaneously. It will be presented as a composite precipitate with staggered Zr and Sc atoms, which is consistent with the experimental phenomenon showing an Sc: Zr ratio at about 1:3 to 1:4 in Al_3_(Zr, Sc) particles in Reference [53]. In addition, based on the lowest interfacial energy of Al/Al_3_Sc, Sc atoms will get priority to take up the interfacial sites and construct the core-shell structure, which is shown in Figure 5a. When the ratio of Sc:Zr is lower than 1:3, redundant Zr atoms will nucleate as part of the Al_3_Zr phase with Al atoms. Since solidification is an unbalanced process, the nucleation of Al_3_Zr(Sc1-1) and Al_3_Zr will form a competitive situation in the actual experiment.

Based on the high absolute value of Al_3_Sc/Al interfacial energy (shown in Table 4) and the high absolute value of Al_3_Zr’s formation enthalpy (shown in Table 2), the core-shell structure with Al_3_Zr-core or Al_3_Zr(Sc1-1)-core sphered with an Sc-rich shell can be formed, as shown in Figure 5. On the other hand, under the condition of an Sc:Zr ratio higher than 1:3, there will also be a competitive nucleation of Al_3_Zr(Sc1-1) and other Al_3_Zr phases based on their high absolute value of formation enthalpy and binding energy and the excessive Sc atoms then form a shell cladding the core. As a result, the core-shell structures of Al_3_(Zr, Sc) are shown in experiments [54].

#### 3.4.2. Griffith Rupture Work and Interfacial Energy of Al(001)/Al_3_(Zr, Sc)(001)

The Griffith rupture work of interfacial models with different doping sites and doping ratios (contrast with Figure 3) is shown in Table 6. From the data, it can be concluded once again that even with Sc doping, interface models with I-ter are more stable than D-ter, which shows that the Griffith rupture works of I-ter models are all higher than that of D-ter ones. Furthermore, the Griffith rupture work of Al/Al_3_Zr (Sc1-1) (I-ter) (shown in Table 6) is higher than both Al/Al_3_Zr(I-ter) and Al/Al_3_Sc(I-ter) (shown in Table 4). This is due to the low interfacial energy of Al/Al_3_Sc (shown in Table 4), the low surfacial energy of Al_3_Sc (shown in Table 4), and the high binding energy of Al_3_Zr (shown in Table 2). All these factors effectively lead to the beneficial effect of Sc doping on the improvement of the interface bond strength. As a result, the sites neighboring the interface are liable to be occupied by Sc atoms. However, the Griffith rupture work shows weak differences in the various doping sites in the interfacial modes, which just show the bonding strength of the interfaces. It can also be summarized that the ratio of Sc: Zr being higher than 1:3 does little good to the interface strengthening. Consequently, Sc-doped Al_3_(Zr, Sc) with the Sc: Zr ratio at 1:3 is beneficial to its nucleation as a grain refiner and as an improvement of interface strength by combining the property of Al_3_(Zr, Sc) (shown in Table 5) with the interfacial property of Al/Al_3_(Zr, Sc) (shown in Table 6).

### 3.5. Electronic Structure

#### 3.5.1. Density of States

**Figure 6:**
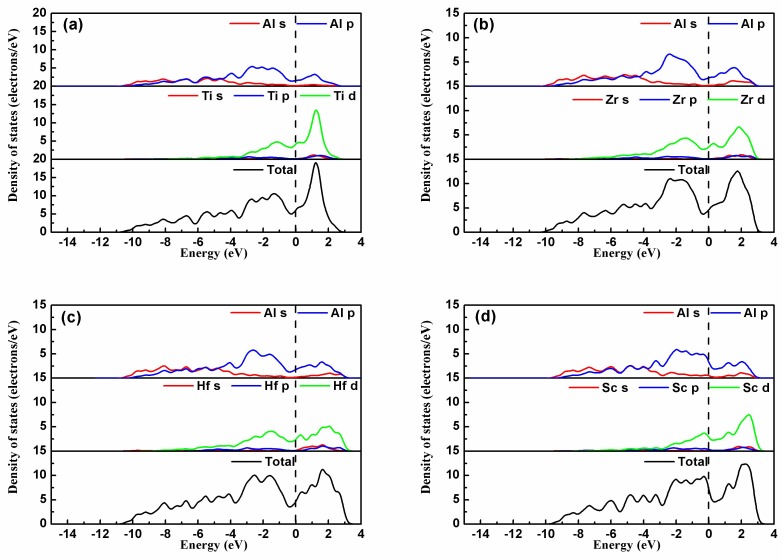
The partial density of states of (**a**) Al_3_Ti, (**b**) Al_3_Zr, (**c**) Al_3_Hf, and (**d**) Al_3_Sc.

For a deeper insight into the electron interaction and the atomic bonding, the partial density of states (PDOS) of the four intermetallics Al_3_TM(TM = Ti, Zr, Hf, Sc) are shown in Figure 6. The dashed line represents the Fermi level. Figure 6 shows that the bonding between Al-TM atoms in the Al_3_TM block is mainly metallic bonding due to numerous valence electrons at the Fermi energy level *E*_F_. In Al_3_Zr, a broad overlap between Zr and Al valence electrons below *E*_F_ indicates that covalent bonding exists between Al-Zr atoms. This means that the binding strength for Al_3_Zr is mainly dominated by metallic bonding, while an influence of covalent bonding between Al-Zr atoms cannot be ignored besides metallic bonding. The PDOS of the other Al_3_TM(Ti, Hf, Sc) phases shows that the electronic interaction between Al-TM(Ti, Hf, Sc) is also mixed with metallic and covalent bonding. However, compared with Al_3_Zr, the overlap between TM(Ti, Hf, Sc) and Al valence electrons below *E*_F_ is weaker than Zr-Al atoms at different degrees, which provides an explanation for the higher binding energy of Al_3_Zr shown in Table 2.

#### 3.5.2. Planar-Averaged Difference Charge

**Figure 7:**
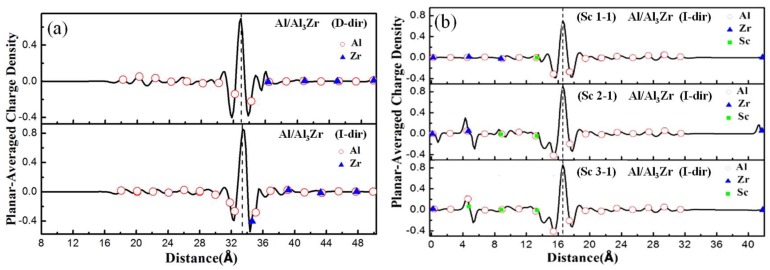
Planar-averaged differential electron density. (**a**) Al/Al_3_Zr interfaces with D-ter and I-ter, (**b**) Al/Al_3_(Zr, Sc) interfaces with different Sc-doping ratio.

Planar-averaged difference charge (relative to the isolated surfaces) for the interface models with different doping ratios and doping sites is shown in Figure 7. Planar-averaged charge is obtained by adding the total electron density in the same atomic layer. Planar-averaged difference charge can be calculated by using Equation (16).

(16) $$\Delta \rho =\rho_{\mathrm{Al}-{\mathrm{Al}}_{3}\left(\mathrm{Zr},\mathrm{Sc}\right)}-\rho_{\mathrm{Al}}-\rho_{{\mathrm{Al}}_{3}\left(\mathrm{Zr},\mathrm{Sc}\right)}$$

Here, $\rho_{\mathrm{Al}-{\mathrm{Al}}_{3}\left(\mathrm{Zr},\;\mathrm{Sc}\right)}$ presents the total electron density of interface model, *ρ*_Al_ is the total electron density of only Al matrix with the Al_3_(Zr, Sc) in the former interface substituted with vacuum, and $\rho_{{\mathrm{Al}}_{3}\left(\mathrm{Zr},\;\mathrm{Sc}\right)}$ represents the total electron density of only Al_3_(Zr, Sc) with the Al matrix in the former interface substituted with vacuum. To ensure the same points of electron density values are obtained, the same cutoff energy and *k*-points must be set during the calculation process.

The planar-averaged charge density shows that the charge transfers around the interface between the Al matrix and Al_3_TM, which is shown in Figure 7. Furthermore, the charge transfer affects the electron distribution of the atoms in two to three layers extending into both sides from the interface. It is clearly shown in Figure 7 that the atoms near the D-ter interfaces move away from their original sites in the interface and bring in lattice distortion. But the atoms in the I-ter interfaces keep the original lattice positions and cause little distortion. So, the interface with I-ter can maintain a better coherent lattice relationship and results in a lower interfacial energy, which makes the I-ter interface more likely to form and explains the inner reason for the results discussed in Section 3.3. Overall, the localization of charge transfer of the Al/Al_3_Zr(I-ter) interface is clearer than that of the Al/Al_3_Zr(D-ter) interface, which shows a higher interface bonding strength. Additionally, the charge transfer distribution in the Al/Al_3_Zr(I-ter) interface model broadens and affects the electron distribution around the inner atoms extending to both sides, which results in more stable I-ter interfaces with higher Griffith rupture work.

Figure 7b shows that the doped Sc atoms increase the interfacial interaction shown by the planar-averaged difference charge. However, the charge transfer of Al/Al_3_Zr (Sc2-1) (I-ter) and Al/Al_3_Zr (Sc3-1) (I-ter) shows little difference from that of Al/Al_3_Zr (Sc1-1) (I-ter). It can be concluded that on the condition that the ratio of Sc:Zr reaches 1:3, after the Al_3_Sc(Zr, Sc):Zr reaches the 1:3 proportion, further increasing of the ratio of Sc:Zr is fruitless for improving the interface bonding strength. In addition, as long as the Zr atom adjacent to the interface is replaced with Sc, the atomic lattices are all coherent with the Al matrix. This is consistent with the results of Griffith rupture work shown in Table 6.

Valence charge density is averaged on each (001) plane. The supercell containing 12 layers of Al_3_(Zr, Sc) and eight layers of Al with a vacuum region is shown. Red circle, blue triangle, and solid green circle indicate the positions of Al, Zr, and Sc in atomic layers, respectively. The interface is marked by a vertical line.

## 4. Conclusions

In summary, we have explored the energy, elastic properties, and electronic structure of Al_3_TM(TM = Ti, Zr, Hf, Sc) and Al_3_(Zr, Sc). Besides, the interfacial properties of Al/Al_3_TM(TM = Ti, Zr, Hf, Sc) and Al/Al_3_(Zr, Sc) have also been investigated by first-principle simulations. Al_3_(Zr, Sc) with the ratio of Sc:Zr at 1:3 shows the highest absolute value of formation enthalpy, which means that it is the most suitable for use as a grain refiner among all the intermetallics. According to elastic properties and their anisotropy characteristics, Sc-doped Al_3_(Zr, Sc) shows a more beneficial isotropy than Al_3_Sc.

Comparing interfaces with two different terminals, Al/Al_3_TM interfaces with I-ter modes are more stable than Al/Al_3_TM interfaces with D-ter modes, because Al/Al_3_TM interfaces with I-ter modes overall show larger Griffith rupture work and a smaller interface energy than Al/Al_3_TM interfaces with D-ter modes. Among all the Al/Al_3_TM(TM = Ti, Zr, Hf, Sc) interfacial modes, the Al/Al_3_Sc interface shows the highest Griffith rupture work and lowest interfacial energy, which indicates that Sc atoms should get priority for occupying interfacial sites. What is more, the binding strength of Al_3_Zr with the Al matrix can effectively be improved by Sc-doping.

## Figures and Tables

**Table 1:** Lattice parameters of the calculated Al_3_TM (TM = Ti, Zr, Hf, Sc) phases.

Phases	Al			Al_3_Ti			Al_3_Zr			Al_3_Hf			Al_3_Sc		
	Cal. [This Work]	Cal.	Exp.	Cal. [This Work]	Cal.	Exp.	Cal. [This Work]	Cal.	Exp.	Cal. [This Work]	Cal.	Exp.	Cal. [This Work]	Cal.	Exp.
Crystal System	Cubic	Cubic	Cubic	Tetragonal	Tetragonal	Tetragonal	Tetragonal	Tetragonal	Tetragonal	Tetragonal	Tetragonal	Tetragonal	Tetragonal	Tetragonal	Tetragonal
Space Group	FM-3M	FM-3M	FM-3M	I4/MMM	I4/MMM	I4/MMM	I4/MMM	I4/MMM	I4/MMM	I4/MMM	I4/MMM	I4/MMM	I4/MMM	I4/MMM	I4/MMM
a = b(Å) (deviation)	4.053	4.046 [12]	4.049 [12]	3.907 (−3.60%)	3.885 [13] 3.81 [14]	3.89 [15]	4.028 (−0.62%)	3.999 [16] 4.008 [17] 4.02 [18]	4.007 [19] 3.999 [20]	4.004 (–1.22%)	3.990 [17] 3.987 [21]	4.01 [21]	4.055 (0.04%)		
c(Å)	4.053	4.046 [12]	4.049 [12]	16.714	16.823 [13] 16.459 [14]	16.922 [15]	17.384	17.283 [16] 17.297 [17] 17.36 [18]	17.286 [19] 17.283 [20]	17.224	17.172 [17] 17.179 [21]	17.653 [21]	17.283		
V(Å^3^)	66.59			255.18			282.10	276.43 [16]		276.09			284.16		

**Table 2:** Enthalpy of the Al_3_TM(TM = Ti, Zr, Hf, Sc) phases in the primitive units and the formation enthalpy and binding energy of the calculated intermetallics.

Phases	Al_3_Ti			Al_3_Zr			Al_3_Hf			Al_3_Sc		
	Cal. [This Work]	Cal.	Exp.	Cal. [This Work]	Cal.	Exp.	Cal. [This Work]	Cal.	Exp.	Cal. [This Work]	Cal.	Exp.
**ΔH** (**eV**/atom)	−0.44			−0.53			−0.44			−0.45	−0.45 [9]	−0.45 [34]
**ΔH** (kJ/mol)	−42.39	−38.90 [16] −41.45 [35] −41.90 [36] −39.51 [37]	−39.2 [38]	−50.65	−49.11 [16] −51.06 [13] −53.45 [39]	−49 ± 4 [40] −48.4 ± 1.3 [41]	−42.85	−39.63 [16] −40.00 [20]	−40. 6 ± 0.8 [42] −44.7 ± 2.4 [43]	−43.41		
**E_b_** (**eV**/atom)	4.524			5.157			4.107			4.048		
**E_b_** (kJ/mol)	436.27			495.85			396.07			423.80		

**Table 3:** Calculated bulk modulus *B* (*GPa*), shear modulus *G* (*GPa*), Young’s modulus *E* (*GPa*), *B/G*, Poisson’s ratio *ν*, and Debye temperature *Θ_D_*(*K*) of the Al_3_TM (TM = Ti, Zr, Hf, Sc) phases.

Phases	Al_3_Ti		Al_3_Zr		Al_3_Hf		Al_3_Sc		Al_12_Zr_3_Sc
	This Work	Other Work	This Work	Other Work	This Work	Other Work	This Work	Other Work	This Work
B_V_	102.4		100.8		106.5		92.1		96.7
B_R_	102.2		100.6		106.0		91.8		96.5
B	102.3	102 [45] 103 [5] 107 [46]	100.7	105.3 [46] 102.2 [47]	106.3	108.2 [46]	91.9	91.8 [48]	96.6
G_V_	81.8		84.6		77.8		74.1		79.2
G_R_	81.7		83.3		77.4		71.7		77.0
G	81.8	88.5 [46]	84.0	83.2 [46] 85.1 [47]	77.6	80.3 [46]	72.9	71.7 [48]	78.1
E	193.8	208.5 [46]	197.2	197.6 [46] 201.8 [47]	187.2	193.3 [46]	173.1		184.6
B/G	1.25	1.22 [48] [46]	1.20	1.26 [46,47]	1.37	1.35 [46]	1.26		1.24
ν	0.18		0.17		0.21		0.19		0.18

**Table 4:** Griffith rupture work (*W*_ad_), surface energy (*σ*_suf_), and interfacial energy (*γ*_int_) of Al_3_TM(001)/Al(001) interfaces (σ_suf(Al)_ = 1.038 (J/m^2^)).

Interfaces	Stacking Site	Al_3_Ti(001)/Al(001)		Al_3_Zr(001)/Al(001)		Al_3_Hf(001)/Al(001)		Al_3_Sc(001)/Al(001)	
Type		D-ter	I-ter	D-ter	I-ter	D-ter	I-ter	D-ter	I-ter
*W*_ad_(J/m^2^)	Top	2.610	2.877	2.783	2.894	2.752	2.886	2.491	2.985
	Bridge	-	-	-	-	-	-	-	-
	Central	-	2.450	-	2.772	-	2.758	-	2.740
σ_suf_(J/m^2^)	Al_3_TM	2.030	1.923	1.824	1.751	1.835	1.780	1.445	1.444
γ_int_(J/m^2^)	Top	0.458	0.084	0.079	−0.105	0.121	−0.068	−0.008	−0.503
	Bridge	-	-	-	-	-	-	-	-
	Central	-	0.511	-	0.017	-	0.06	-	−0.258

“-” indicates instability. Atoms in bridge-site stacking modes all shift to top or central sites after geometry optimization. Central-site stacking modes with D-ter types are also partially unstable, which shows that atoms move to top sites after geometry optimization.

**Table 5:** Formation enthalpy and binding energy of Al_3_(Zr,Sc).

Phases	Δ*H*(eV/atom)	Δ*H* (kJ/mol)	*E*_b_(eV/atom)	*E*_b_ (kJ/mol)
Al_3_Zr (Sc1-1)	−0.532	−51.14	−4.996	−480.35
Al_3_Zr (Sc2-1)	−0.504	−48.44	−4.799	−461.42
Al_3_Zr (Sc2-2)	−0.509	−48.98	−4.804	−461.96
Al_3_Zr (Sc2-3)	−0.509	−48.98	−4.804	−461.96
Al_3_Zr (Sc3-1)	−0.481	−46.27	−4.608	−443.03
Al_3_Sc	−0.450	−43.27	−4.408	−423.80
Al_3_Zr	−0.524	−50.42	−5.157	−495.85

**Table 6:** Griffith separation work of interface models with different Sc-doped sites and Sc-doping ratios.

Model	*W*ad (J/m^2^)	Model	*W*ad (J/m^2^)	Model	*W*ad (J/m^2^)
Al/Al_3_Zr (Sc1-1) (I-ter)	3.108	Al/Al_3_Zr (Sc2-1) (I-ter)	2.998	Al/Al_3_Zr (Sc3-1) (I-ter)	3.008
Al/Al_3_Zr (Sc1-2) (I-ter)	2.996	Al/Al_3_Zr (Sc2-2) (I-ter)	3.000	Al/Al_3_Zr (Sc3-2) (I-ter)	3.014
Al/Al_3_Zr (Sc1-3) (I-ter)	3.022	Al/Al_3_Zr (Sc2-3) (I-ter)	3.021	Al/Al_3_Zr (Sc3-3) (I-ter)	3.021
		Al/Al_3_Zr (Sc2-4) (I-ter)	3.008	Al/Al_3_Zr (Sc3-4) (I-ter)	3.014
Al/Al_3_Zr(Sc1-1) (D-ter)	2.501	Al/Al_3_Zr(Sc2-1) (D-ter)	2.502	Al/Al_3_Zr(Sc3-1) (D-ter)	2.498
Al/Al_3_Zr(Sc1-2) (D-ter)	2.825	Al/Al_3_Zr(Sc2-2) (D-ter)	2.532	Al/Al_3_Zr(Sc3-2) (D-ter)	2.485
Al/Al_3_Zr(Sc1-3) (D-ter)	2.829	Al/Al_3_Zr(Sc2-3) (D-ter)	2.886	Al/Al_3_Zr(Sc3-3) (D-ter)	2.503
		Al/Al_3_Zr(Sc2-4) (D-ter)	2.503	Al/Al_3_Zr(Sc3-4) (D-ter)	2.838
